# A successful start for *mLife*

**DOI:** 10.1002/mlf2.12061

**Published:** 2023-03-20

**Authors:** Li Huang, Jizhong Zhou

**Affiliations:** ^1^ State Key Laboratory of Microbial Resources, Institute of Microbiology Chinese Academy of Sciences Beijing China; ^2^ College of Life Sciences University of Chinese Academy of Sciences Beijing China; ^3^ Institute for Environmental Genomics University of Oklahoma Norman OK USA; ^4^ Department of Microbiology and Plant Biology University of Oklahoma Norman OK USA; ^5^ School of Civil Engineering and Environmental Sciences University of Oklahoma Norman OK USA; ^6^ School of Computer Science University of Oklahoma Norman OK USA; ^7^ Earth and Environmental Sciences Lawrence Berkeley National Laboratory Berkeley CA USA

*mLife* made its debut with the publication of the inaugurating issue on March 30, 2022. We are pleased to have published 42 high‐quality articles, including reviews, research articles, and application notes, which cover microbial genetics, biochemistry and physiology, ecology and evolution, pathogenic and public health microbiology, and so forth. Nine of the articles concern technical innovations and applications in areas including genome editing, single‐cell analysis, and database. To pay tribute to the great microbiologist Carl Woese on the 10th anniversary of his death, the Journal published a special section containing three thought‐provoking articles in the last issue of the year. *mLife* and its collaborating publisher Wiley have made great efforts to increase the Journal's national and continental representation. As a result, the Journal received submissions from over 20 countries in five continents last year. The authors of the published articles are from China, United States, Great Britain, France, Canada, Germany, Japan, Switzerland, and Austria.

We believe that quality is the life of a journal, and have strived to uphold the highest academic standard for *mLife*. Last year, we implemented strict and professional review and editing policies. Our editorial board members and invited reviewers have done a great job to ensure that only high‐quality studies in a given field of microbiology were considered. As a result, only about 13% of the free submissions were accepted. Although it is apparently too early to provide a reliable assessment of the impacts of *mLife* as well as articles published so far in the journal, *mLife* fares relatively well among newly launched journals in microbiology and attracted a quite broad audience based on various metrics. Over 35,000 downloads from 39 countries were recorded for the last nine months of 2022 by Wiley Online Library alone.

To serve the microbiology community and increase the visibility of *mLife*, we started a webinar series, referred to as the *mLife* Forum, which generally included three invited talks for a total of about two hours for each webinar. Five webinars on various topics of current interest were successfully held last year, often attracting a large audience (>3000 attendants/webinar). In collaboration with the Chinese Society for Microbiology (CSM), we have also incorporated a forum for early‐career microbiologists into the regular program of the annual conference of CSM in 2022. The early career speakers were selected based on their excellent research published in *mLife* by associate editors and editors‐in‐chief.

In its pursuit of excellence, *mLife* will strive to be innovative in scientific publication and further improve the editorial services to our authors by offering rapid and professional peer review, high‐level copyediting and figure polishing, and timely and wide dissemination at no publication charge. The Journal will also make efforts to raise the number of articles published per year and increase the representation of countries in the publication.

The new journal has received enthusiastic support from colleagues around the world, to whom we are deeply indebted. Also, we would like to express our appreciation to all editorial board members, authors, reviewers as well as Wiley for their enormous contributions to the publication of *mLife*. After the successful 1st year, we are confident that *mLife* will become a top‐tier journal in the field of microbiology in the near future.

